# Healthy aging and quality of life in University of the Third Age students versus community peers: a cross-sectional study

**DOI:** 10.1186/s12877-026-08514-3

**Published:** 2026-10-14

**Authors:** Derya Bıçak Ayık, Fadime Yel, Derya Akça Doğan

**Affiliations:** 1 Department of Internal Medicine Nursing, Faculty of Health Sciences, Mardin Artuklu University, Mardin, Turkey; 2 Department of Medical Services and Techniques, First and Emergency Aid Program, Alanya Alaaddin Keykubat University, Alanya, Turkey; 3 Department of Internal Medicine Nursing, Faculty of Health Sciences, Bursa Uludag University, Bursa, Turkey

**Keywords:** Healthy aging, Quality of life, Education, continuing, Universities, Aged

## Abstract

**Objective:**

This study aimed to compare healthy aging and quality of life levels between University of the Third Age (U3A) students and older adults not enrolled in the program and to examine the relationship between healthy aging and quality of life.

**Methods:**

This descriptive, analytical, and cross-sectional study included 297 older adults aged 60 years and older (mean age: 68.51 ± 5.21 years): 146 U3A students and 151 older adults not enrolled in the program. Data were collected using the Participant Information Form, the Healthy Aging Instrument (HAI), and the Quality of Life Scale in Older People (CASP-19). Descriptive statistics, the Mann–Whitney U test, chi-square test, Spearman’s correlation analysis, and multiple linear regression analysis were used.

**Results:**

U3A students had significantly higher HAI and CASP-19 total and subscale scores than older adults not enrolled in the program (*p* < 0.05 for all comparisons). A moderate positive correlation was found between healthy aging and quality of life (rho = 0.559, *p* < 0.001). After adjustment for age, gender, marital status, education level, chronic disease status, self-rated health status, cigarette/tobacco use, and regular health check-ups, U3A student status remained independently associated with both healthy aging and quality of life (*p* < 0.001).

**Conclusion:**

U3A students had higher healthy aging and quality of life scores than their peers who were not enrolled in the program, and these differences remained significant after adjustment for sociodemographic and health-related variables. A moderate positive correlation was also found between healthy aging and quality of life. These findings indicate that U3A programs may be considered within the scope of community-based healthy aging initiatives. However, causal inferences cannot be made because of the cross-sectional design.

## Introduction

The global population is aging rapidly, and the proportion of older adults in the total population continues to increase [1, 2]. Increasing life expectancy has made not only extending lifespan but also enabling individuals to live healthy, independent, and productive lives in older age a major priority of global health policies [1, 3]. In line with this priority, the United Nations proclaimed 2021–2030 the Decade of Healthy Ageing, led by the World Health Organization [4]. WHO defines healthy aging as the process of developing and maintaining the functional ability that enables well-being in older age [4]. This approach emphasizes that healthy aging does not merely refer to the absence of disease or disability but is a multidimensional process encompassing the maintenance of physical, cognitive, psychological, and social functioning [2, 5].

Healthy aging is currently recognized as being shaped not only by biological processes but also by lifelong behaviors and environmental conditions [5, 6]. Modifiable factors such as regular physical activity, healthy lifestyle habits, stress management, self-care behaviors, cognitive stimulation, and social participation play an important role in helping older adults maintain functional independence and remain actively engaged in life [1, 5–8]. Social determinants, including education level, lifelong learning opportunities, and social support, are also associated with healthy aging. Higher education levels have been linked to more favorable health behaviors, better mental health, lower disease burden, and greater life satisfaction [8, 9].

Quality of life is considered one of the main indicators of healthy aging. WHO defines quality of life as individuals’ perceptions of their lives within the context of their culture, values, expectations, and personal goals [2, 8]. Among older adults, quality of life is shaped by the interaction of physical health, psychological well-being, independence, social relationships, and environmental conditions [5, 6]. Therefore, quality of life is considered not only an outcome of healthy aging but also a key indicator reflecting functional capacity and overall well-being. Studies have shown that older adults with a higher quality of life have lower frailty and greater functional capacity and life satisfaction [1, 10, 11]. Conversely, social isolation, low physical activity, cognitive decline, and psychological problems are among the main factors associated with poorer quality of life [1, 11–13].

In recent years, lifelong learning and social participation have been recognized as important modifiable factors supporting healthy aging. Social participation not only helps older adults maintain their connections with society but may also contribute to preserving physical, cognitive, and psychological capacities [2]. Participation in educational, cultural, and social activities may support cognitive functioning, strengthen social relationships, and promote life satisfaction among older adults. Studies have shown that socially active older adults have a higher quality of life, better psychological well-being, more favorable self-rated health, and greater life satisfaction [14, 15]. Volunteering, regular participation in social activities, and strong social networks have also been associated with less loneliness, depression, and anxiety and with better quality of life [16, 17].

One of the most prominent models supporting lifelong learning is the University of the Third Age (U3A). Implemented in many countries, this model aims to support active aging by encouraging older adults to participate in educational, cultural, artistic, physical, and social activities [18, 19]. U3A models vary across countries in their organizational structure and approach. While some U3A models are university-based, the United Kingdom model is more community-based and self-directed, with members teaching and learning from one another [20]. U3A programs are not merely educational initiatives focused on acquiring knowledge; they are multicomponent lifelong learning environments that support social interaction, cognitive stimulation, self-efficacy, active citizenship, and community integration [8, 21]. Previous studies have reported higher quality of life, self-rated health, social participation, and psychological well-being among older adults participating in these programs [9, 22, 23]. A recent quasi-experimental study conducted in Türkiye also reported significant improvements in quality of life, life satisfaction, and successful aging following participation in a U3A program [24].

Nevertheless, important gaps remain in the literature. Most studies have examined quality of life, life satisfaction, or successful aging separately, whereas studies evaluating healthy aging and quality of life together remain limited. Similarly, few studies have directly compared U3A students with community-dwelling older adults who are not enrolled in these programs. The potential role of participation in lifelong learning programs in the association between healthy aging and quality of life also remains insufficiently understood. Comparative studies are therefore needed to clarify how lifelong learning-based initiatives are associated with healthy aging and quality of life.

This study aimed to compare healthy aging and quality of life levels between older adults who were U3A students and those who were not enrolled in the program. It also examined the relationship between healthy aging and quality of life and assessed whether the between-group differences persisted after accounting for sociodemographic and health-related variables. We hypothesized that U3A students would have higher healthy aging and quality of life levels than their peers who were not enrolled in the program and that healthy aging would be positively correlated with quality of life.

## Materials and methods

### Study design

This descriptive, analytical, and cross-sectional study was conducted to compare healthy aging and quality of life levels between older adults who were U3A students and those who were not enrolled in the program. The relationship between healthy aging and quality of life was also examined, and whether U3A student status was independently associated with these two outcomes was evaluated. The study was reported in accordance with the Strengthening the Reporting of Observational Studies in Epidemiology (STROBE) guidelines.

### Setting and participants

The study was conducted between March and December 2025 in Alanya, Antalya, Türkiye. The U3A curriculum at Alanya Alaaddin Keykubat University is structured as a four-year program, with fall and spring semesters in each academic year. The university-based program includes health education, physical activity and sports, skills-based courses, cultural activities, and elective courses. Data from the U3A and comparison groups were collected concurrently during this period. The study group consisted of older adults enrolled in the 60 + U3A program, whereas the comparison group consisted of older adults living in the same region who were not enrolled in the program.

Individuals aged 60 years or older who could read and understand Turkish and voluntarily agreed to participate were eligible for inclusion. For the comparison group, an additional eligibility criterion was that participants were not currently enrolled in the U3A program or any other organized lifelong learning program. The presence of Alzheimer’s disease, dementia, or cognitive impairment severe enough to prevent participants from answering the study questions was an exclusion criterion; however, no participants were excluded for these reasons. Forms with missing responses were excluded during data cleaning.

**Fig. 1:**
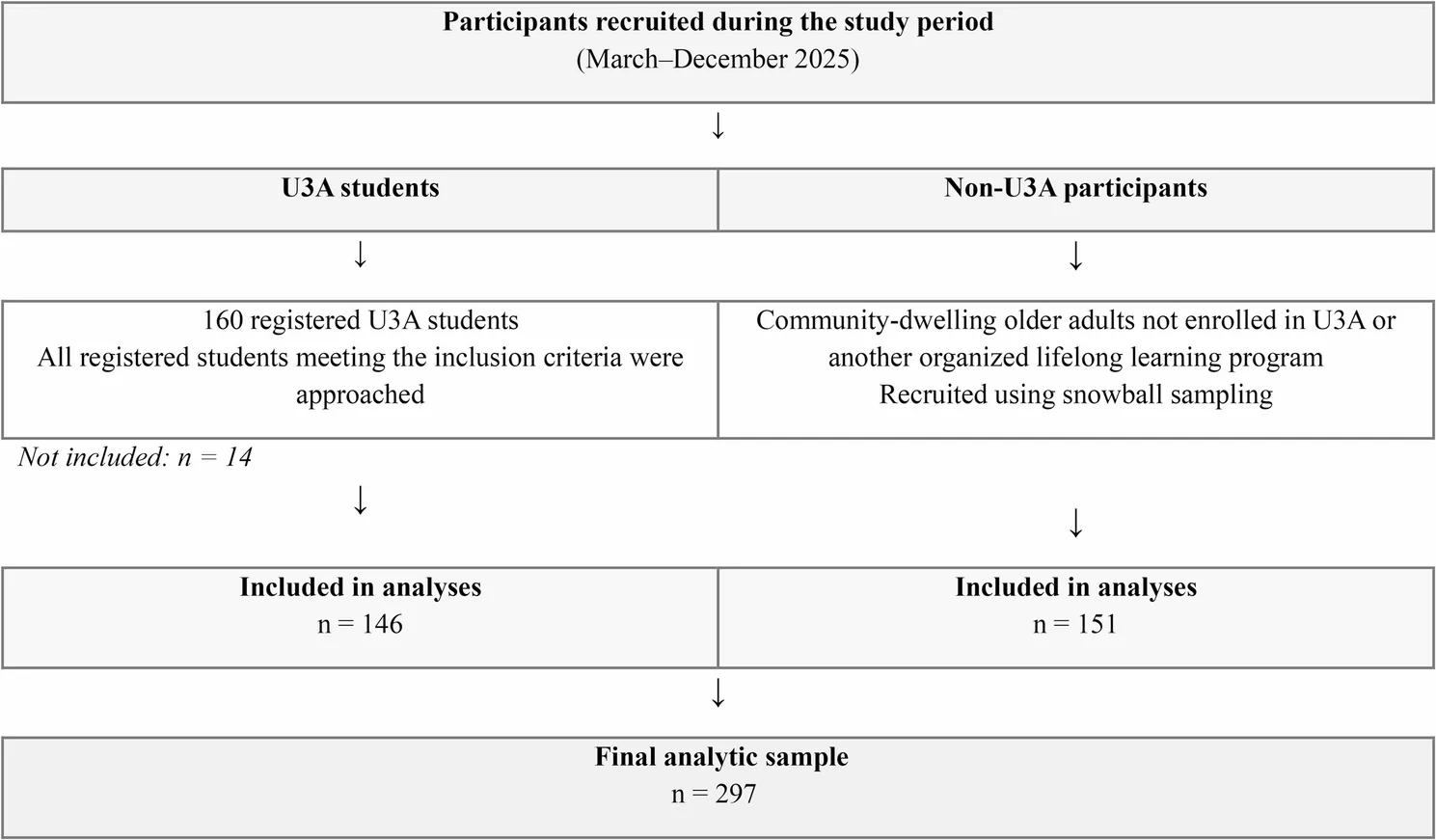
Flow diagram of participant selection during the study period

No probability sampling method was used. A census approach was adopted for the U3A group, whereas snowball sampling was used for the comparison group. During the study period, 160 students were enrolled in the Alanya Alaaddin Keykubat University 60 + U3A program, including 60 first-year, 55 s-year, and 45 third-year students. All registered students who met the inclusion criteria were approached. Of the 160 registered students, 146 were included in the analyses (91.3%). Initial participants in the comparison group were reached through local social networks, after which snowball sampling continued through participant referrals to other eligible individuals. Only individuals who voluntarily agreed to participate were included in either group. The sample size was not based on an a priori calculation; instead, eligible volunteers who could be reached during the study period were recruited. Following data cleaning, the analyses included 297 participants: 146 U3A students and 151 older adults not enrolled in the program. The participant selection process is summarized in Fig. 1.

### Data collection instruments

Data were collected using the Participant Information Form, the Healthy Aging Instrument (HAI), and the Quality of Life Scale in Older People (CASP-19). The HAI and CASP-19 were selected because they closely aligned with the aims of this study and the key constructs being examined. The HAI assesses multiple dimensions of healthy aging, including physical activity, social relationships and participation, cognitive activity, and self-care. The CASP-19 was considered particularly appropriate because it was specifically developed to assess quality of life in older adults.

#### Participant information form

The participant information form, developed by the researchers based on the literature, consisted of 13 questions assessing participants’ sociodemographic and health-related characteristics. The form included age, gender, marital status, education level, employment status, income level, presence of chronic disease, self-rated health status, cigarette/tobacco use, alcohol use, and regular health check-ups. In addition, U3A student status was assessed, and year of study was recorded for U3A students. Whether participants had previously received formal university education or education through a U3A program was also recorded.

#### Healthy aging instrument (HAI)

The Healthy Aging Instrument was developed by Thiamwong et al. [25], and its Turkish validity and reliability study was conducted by Yıldırım et al. The original scale is a 5-point Likert-type scale consisting of 35 items and 9 factors. In the Turkish validity and reliability study, the scale was adapted as a 31-item structure with eight subscales: Staying physically active, Having social relationships and support, Managing stress, Accepting aging and being self-sufficient, Practicing self-care and self-awareness, Making good deeds, Staying cognitively active and having social participation, and Living simply and making merit. The Turkish version contains no reverse-scored items. The total score of the Turkish version ranges from 31 to 155. Higher scores on the total scale and its subscales indicate a higher level of healthy aging practices. The internal consistency of the Turkish version of the HAI was reported as 0.88 [26], while it was found to be 0.95 in the present study.

#### Quality of life scale in older people (CASP-19)

The CASP-19 was developed by Hyde et al. [27], and its Turkish validity and reliability study was conducted by Türkoğlu and Adıbelli. The Turkish form was structured as a 13-item scale with two factors: Perception of autonomy and satisfaction and Perception of barriers. Each item is rated on a 4-point scale: 0 = never, 1 = rarely, 2 = sometimes, and 3 = always. The total score ranges from 0 to 39, with higher scores indicating a higher quality of life [28]. Higher scores on the perception of barriers subscale indicate fewer perceived barriers. The internal consistency coefficient of the Turkish 13-item version of the CASP-19 was reported as 0.91, while it was found to be 0.88 in the present study.

### Data collection process

Following ethics committee approval, participants were informed of the study’s purpose and scope, and their written informed consent was obtained. Data were collected from all participants through face-to-face interviews; interviews with U3A students were conducted on class days. The researchers administered the data collection instruments, and participants’ responses were recorded electronically using Google Forms. Each interview lasted approximately 20–25 min. All participants received the same standardized explanations, and no guidance that could influence their responses was provided.

### Statistical analysis

Data were analyzed using IBM SPSS Statistics for macOS, version 28.0.0.0 (IBM Corp., Armonk, NY, USA). Descriptive statistics were used to summarize the sociodemographic, health-related, and scale variables. Continuous variables were presented as mean±standard deviation and/or median (Q1–Q3), as appropriate, whereas categorical variables were presented as frequency and percentage. The distribution of continuous variables was examined using graphical methods and normality tests before comparative analyses.

Sociodemographic and health-related characteristics were compared between U3A students and non-U3A participants using the independent samples t-test for age and the chi-square test for categorical variables. As the HAI and CASP-19 total and subscale scores showed departures from normality, between-group comparisons of HAI and CASP-19 scores were conducted using the Mann–Whitney U test. Mean ± standard deviation and median (Q1–Q3) were both provided descriptively, whereas between-group p values were based on the Mann–Whitney U test. Exploratory comparisons of HAI and CASP-19 total scores by year of study among U3A students were performed using the Kruskal–Wallis test. Associations between HAI and CASP-19 scores were examined using Spearman’s correlation analysis.

To evaluate whether U3A student status was independently associated with healthy aging and quality of life, multiple linear regression analyses were performed. Separate adjusted models were constructed for HAI total score and CASP-19 total score. Both models included U3A student status as the main independent variable and were adjusted for age, gender, marital status, education level, chronic disease status, self-rated health status, cigarette/tobacco use, and regular health check-ups. Multicollinearity was assessed using tolerance and variance inflation factor (VIF) values. Internal consistency of the scales was evaluated using Cronbach’s alpha. All statistical tests were two-tailed, and *p* < 0.05 was considered statistically significant.

### Ethical considerations

The study was conducted in accordance with the principles of the Declaration of Helsinki. Ethical approval was obtained from the Alanya Alaaddin Keykubat University Non-Interventional Clinical Research Ethics Committee (Decision No. 02/10; February 11, 2025). Written informed consent was obtained from all participants before the study. Participants were informed that participation was entirely voluntary and that they could withdraw from the study at any time without providing a reason. All data were analyzed anonymously, and participant confidentiality was maintained throughout the study.

## Results

### Characteristics of older adults according to U3A student status

A total of 297 older adults aged 60 years and older were included in the study, comprising 146 U3A students and 151 non-U3A participants (Table 1). Among U3A students, 37.0% (*n* = 54) were first-year, 33.6% (*n* = 49) were second-year, and 29.5% (*n* = 43) were third-year students.

**Table 1:** Sociodemographic and health-related characteristics of older adults according to U3A student status

Characteristics		Total (*n* = 297)	U3A students (*n* = 146)	Non-U3A participants (*n* = 151)	*p*-value
Age, mean ± SD		68.51 ± 5.21	68.62 ± 5.02	68.40 ± 5.39	0.717ᵃ
Gender, n (%)	Male	87 (29.3)	35 (24.0)	52 (34.4)	**0.048ᵇ**
	Female	210 (70.7)	111 (76.0)	99 (65.6)	
Marital status, n (%)	Married	201 (67.7)	96 (65.8)	105 (69.5)	0.486ᵇ
	Single	96 (32.3)	50 (34.2)	46 (30.5)	
Education, n (%)	High school or below	165 (55.6)	76 (52.1)	89 (58.9)	0.233ᵇ
	University or above	132 (44.4)	70 (47.9)	62 (41.1)	
Employment status, n (%)	Employed	24 (8.1)	15 (10.3)	9 (6.0)	0.173ᵇ
	Unemployed	273 (91.9)	131 (89.7)	142 (94.0)	
Income level, n (%)	Income < expenses	40 (13.5)	18 (12.3)	22 (14.6)	0.787ᵇ
	Income = expenses	194 (65.3)	98 (67.1)	96 (63.6)	
	Income > expenses	63 (21.2)	30 (20.5)	33 (21.9)	
Presence of chronic disease, n (%)	Yes	146 (49.2)	79 (54.1)	67 (44.4)	0.093ᵇ
Self-rated health status, n (%)	Good	173 (58.2)	95 (65.1)	78 (51.7)	**0.019ᵇ**
	Moderate	124 (41.8)	51 (34.9)	73 (48.3)	
Cigarette/tobacco use, n (%)	Yes	47 (15.8)	18 (12.3)	29 (19.2)	0.105ᵇ
Alcohol use, n (%)	Yes	32 (10.8)	11 (7.5)	21 (13.9)	0.077ᵇ
Regular health check-ups, n (%)	Yes	256 (86.2)	132 (90.4)	124 (82.1)	**0.038ᵇ**

ᵃIndependent samples t-test, ᵇChi-square test. Bold p-values indicate statistical significance (*p* < 0.05)

Abbreviations: SD, standard deviation; U3A, University of the Third Age

The mean age of the participants was 68.51 ± 5.21 years. Overall, 70.7% were female, 67.7% were married, 44.4% had a university degree or above, and 49.2% reported at least one chronic disease. Most participants were unemployed (91.9%), and 86.2% reported regular health check-ups (Table 1).

No significant differences were observed between U3A students and non-U3A participants in terms of age, marital status, education level, employment status, income level, chronic disease status, cigarette/tobacco use, or alcohol use (*p* > 0.05). However, the groups differed significantly by gender, self-rated health status, and regular health check-ups. The proportion of women was higher among U3A students (*p* = 0.048). A higher proportion of U3A students reported good self-rated health (*p* = 0.019) and regular health check-ups (*p* = 0.038) (Table 1).

### Healthy aging and quality of life according to U3A student status

The mean HAI total score was 119.46 ± 19.34. U3A students had significantly higher mean HAI total scores than non-U3A participants (125.58 ± 13.53 vs. 113.54 ± 22.12; *p* < 0.001) (Table 2). Significant between-group differences were observed across all HAI subscales, with higher scores among U3A students in physical activity, social relationships and support, stress management, acceptance of aging and self-sufficiency, self-care and self-awareness, making good deeds, cognitive activity and social participation, and living simply and making merit (Table 2).

**Table 2:** Comparison of HAI and CASP-19 scores according to U3A student status

Scale scores	Total (*n* = 297)		U3A students (*n* = 146)		Non-U3A participants (*n* = 151)		
	Mean ± SD	Median (Q1–Q3)	Mean ± SD	Median (Q1–Q3)	Mean ± SD	Median (Q1–Q3)	*p*-value
**HAI total score**	119.46 ± 19.34	121 (113–130)	125.58 ± 13.53	125 (116.75–134)	113.54 ± 22.12	118 (106–126)	**< 0.001**
Staying physically active	22.72 ± 4.64	24 (20–26)	24.12 ± 3.62	24 (22–26)	21.38 ± 5.11	22 (18–24)	**< 0.001**
Having social relationships and support	19.48 ± 3.88	20 (18–22)	20.55 ± 2.87	20 (19–23)	18.44 ± 4.41	20 (17–20)	**< 0.001**
Managing stress	13.88 ± 3.53	14 (12–16)	14.40 ± 3.26	15 (12–16)	13.38 ± 3.71	14 (11–16)	**0.040**
Accepting aging and being self-sufficient	15.61 ± 2.72	16 (14–17)	16.23 ± 2.09	16 (15–17)	15.01 ± 3.11	16 (14–16)	**< 0.001**
Practicing self-care and self-awareness	12.19 ± 2.24	12 (12–14)	12.76 ± 1.78	12 (12–14)	11.64 ± 2.49	12 (11–12)	**< 0.001**
Making good deeds	12.51 ± 2.30	12 (12–14)	13.21 ± 1.75	13 (12–15)	11.83 ± 2.55	12 (12–13)	**< 0.001**
Staying cognitively active and having social participation	11.38 ± 2.56	12 (10–13)	12.16 ± 1.84	12 (11–13)	10.64 ± 2.92	12 (9–12)	**< 0.001**
Living simply and making merit	11.69 ± 2.29	12 (11–13)	12.16 ± 2.09	12 (11–14)	11.23 ± 2.38	12 (10–12)	**< 0.001**
**CASP-19 total score**	29.74 ± 6.66	31 (26–35)	31.96 ± 5.33	33 (28–36)	27.59 ± 7.12	29 (23–33)	**< 0.001**
Perception of autonomy and satisfaction	24.35 ± 5.55	26 (21–29)	25.89 ± 4.47	27 (22.75–30)	22.85 ± 6.08	25 (19–28)	**< 0.001**
Perception of barriers	5.39 ± 2.08	5 (4–7)	6.07 ± 1.81	6 (5–8)	4.74 ± 2.11	5 (3–6)	**< 0.001**

Values are presented as mean ± SD and median (Q1–Q3). Mann–Whitney U test was used for group comparisons

Bold p-values indicate statistical significance (*p* < 0.05)

Abbreviations: HAI, Healthy Aging Instrument; CASP-19, Quality of Life Scale in Older People; SD, standard deviation; U3A, University of the Third Age

The mean CASP-19 total score was 29.74 ± 6.66. Similar to the HAI findings, U3A students had significantly higher mean CASP-19 total scores than non-U3A participants (31.96 ± 5.33 vs. 27.59 ± 7.12; *p* < 0.001). U3A students also had significantly higher scores on both CASP-19 subscales. These results showed higher scores on the perception of autonomy and satisfaction subscale, as well as on the perception of barriers subscale, among U3A students (Table 2). Exploratory analyses among U3A students showed that HAI and CASP-19 total scores did not differ significantly across the first, second, and third years of study (*p* > 0.05 for both).

### Correlations between healthy aging and quality of life

Spearman correlation analysis showed positive correlations between HAI and CASP-19 scores (Table 3). The HAI total score was moderately correlated with the CASP-19 total score (rho = 0.559) and with perception of autonomy and satisfaction (rho = 0.570). A weaker but significant correlation was observed between the HAI total score and perception of barriers (rho = 0.307).

**Table 3:** Correlations between HAI and CASP-19 scores

HAI scores	CASP-19 total score	Perception of autonomy and satisfaction	Perception of barriers
**HAI total score**	0.559**	0.570**	0.307**
Staying physically active	0.487**	0.472**	0.297**
Having social relationships and support	0.499**	0.516**	0.267**
Managing stress	0.370**	0.374**	0.211**
Accepting aging and being self-sufficient	0.185*	0.231**	0.032
Practicing self-care and self-awareness	0.390**	0.389**	0.219**
Making good deeds	0.350**	0.375**	0.198**
Staying cognitively active and having social participation	0.456**	0.443**	0.312**
Living simply and making merit	0.268**	0.287**	0.139*

Values are presented as Spearman’s rho

*p < 0.05, **p < 0.001

Abbreviations: HAI, Healthy Aging Instrument; CASP-19, Quality of Life Scale in Older People

Most HAI subscales were positively correlated with CASP-19 total and subscale scores. The strongest subscale-level correlations with CASP-19 total score were observed for having social relationships and support, staying physically active, and staying cognitively active and having social participation. The only non-significant association was between accepting aging and being self-sufficient and perception of barriers (Table 3).

### Unadjusted and adjusted comparison of HAI and CASP-19 total scores

In the unadjusted analyses, U3A student status was associated with a 12.04-point higher HAI total score (95% CI: 7.83–16.24; *p* < 0.001) and a 4.37-point higher CASP-19 total score (95% CI: 2.93–5.81; *p* < 0.001).

In the multiple linear regression models, U3A student status remained significantly associated with both HAI and CASP-19 total scores in models adjusted for age, gender, marital status, education level, chronic disease status, self-rated health status, cigarette/tobacco use, and regular health check-ups (Table 4). Across both regression models, no multicollinearity was detected, with VIF values ranging from 1.036 to 1.222.

**Table 4:** Unadjusted and adjusted comparison of HAI and CASP-19 total scores according to U3A student status

Variable	Unadjusted B (95% CI)	Unadjusted *p*-value	Adjusted B (95% CI)	β	Adjusted *p*-value	*R*²	Adjusted *R*²
HAI total score	12.039 (7.834 to 16.244)	**< 0.001**	10.218 (5.987 to 14.448)	0.265	**< 0.001**	0.173	0.147
CASP-19 total score	4.370 (2.929 to 5.810)	**< 0.001**	3.406 (2.049 to 4.762)	0.256	**< 0.001**	0.283	0.261

Unadjusted estimates were obtained from separate linear regression models including U3A student status as the only independent variable. Multiple linear regression models were adjusted for age, gender, marital status, education level, chronic disease status, self-rated health status, cigarette/tobacco use, and regular health check-ups. The model for HAI total score was significant (F = 6.656, *p* < 0.001), as was the model for CASP-19 total score (F = 12.615, *p* < 0.001). Reference categories were non-U3A participants, female gender, married status, high school or below, no chronic disease, good self-rated health, non-smoker, and no regular health check-ups. Multicollinearity was not detected in either model, with VIF values ranging from 1.036 to 1.222

Abbreviations: CI, confidence interval; HAI, Healthy Aging Instrument; CASP-19, Quality of Life Scale in Older People; U3A, University of the Third Age; B, unstandardized coefficient; β, standardized regression coefficient; VIF, variance inflation factor

U3A student status was associated with a 10.22-point higher HAI total score (95% CI: 5.987 to 14.448; *p* < 0.001) and a 3.41-point higher CASP-19 total score (95% CI: 2.049 to 4.762; *p* < 0.001). The R² values indicated that the models explained 17.3% of the variance in HAI total scores and 28.3% of the variance in CASP-19 total scores (Table 4).

## Discussion

In this study, older adults who were U3A students had higher healthy aging and quality of life scores than their peers who were not enrolled in the program. These differences remained statistically significant after adjustment for selected sociodemographic and health-related variables. A moderate positive correlation was also found between healthy aging and quality of life.

In addition to the HAI total score, U3A students had higher scores across all HAI subscales. Although previous studies have primarily examined the relationship between lifelong learning and quality of life [1, 17, 22, 29], studies evaluating different components of healthy aging together remain limited. Therefore, the present study contributes to the literature by indicating that U3A participation may be associated not only with one aspect of healthy aging but also with its physical, cognitive, behavioral, and social dimensions. The significant differences observed across all subscales suggest that the association may reflect a multidimensional pattern rather than a single behavioral difference.

The higher scores observed on the Staying Physically Active subscale suggest that U3A students may have been more likely to maintain an active lifestyle than older adults who were not enrolled in the program during the study period. This difference may be related not only to physical activities offered within the program but also to behaviors that increase daily mobility, such as regularly attending the campus, participating in group activities, and engaging more actively in social life. The role of physical activity in maintaining functional capacity, reducing frailty, and supporting independent living is well established [10, 30, 31]. A recent meta-analysis showed that mind–body exercises and regular physical activity were associated with meaningful improvements in quality of life and functional independence among older adults [10]. Similarly, Schwartz et al. (2025) reported that exercise programs improved both physical and mental health-related quality of life [31]. Our findings are consistent with these studies and suggest that participation in lifelong learning programs may be associated with behaviors that support physical activity and healthy aging. However, the observed association may also partly reflect differences in lifestyle characteristics between individuals who chose to participate in the program and those who did not.

The higher scores of U3A students on the Having Social Relationships and Support subscale represent another noteworthy finding, indicating an association between participation in lifelong learning programs and stronger social relationships and support. Social support is considered an important determinant of reduced loneliness, greater psychological resilience, and the maintenance of healthy behaviors among older adults [14, 22]. By regularly bringing together individuals with shared interests, U3A programs may provide opportunities to establish new social networks and strengthen existing relationships. Gonnord et al. (2023) reported that social relationships were an important mechanism through which group-based physical and cognitive activities were associated with better quality of life among older adults [29]. Similarly, Ge et al. (2025) found that participation in volunteer activities strengthened social networks, which was associated with fewer depressive symptoms and greater life satisfaction [14]. Kim and Choi (2024) also reported that regular participation in social activities was associated with better self-rated health [16]. The higher social support scores observed in our study suggest that U3A may serve not only as an educational setting but also as an environment that supports social relationships and participation.

Higher scores among U3A students on the Staying Cognitively Active and Having Social Participation subscales were also noteworthy. The maintenance of cognitive functioning in older age is associated not only with neurological processes but also with environmental factors that provide cognitive stimulation [5]. Activities such as learning new information, participating in discussions, solving problems, attending cultural events, and interacting with peers may contribute to maintaining cognitive reserve [8, 14]. Zhang et al. (2024) reported that perceived educational quality in U3A was associated with greater subjective well-being and that this relationship was explained by the fulfillment of autonomy, competence, and relatedness needs [23]. These findings may provide one possible explanation for the higher cognitive activity and social participation scores observed in our study.

The higher scores of U3A students on the Practicing Self-Care and Self-Awareness and Accepting Aging and Being Self-Sufficient subscales indicate that program participation may also be associated with behavioral and psychological dimensions of healthy aging. Health-related education may increase individuals’ awareness of monitoring their health, appropriately using healthcare services, and maintaining health-promoting behaviors [16, 22, 29]. Interacting with individuals who have similar life experiences may also facilitate acceptance of aging as a natural process and strengthen self-efficacy [9, 23]. Thus, U3A programs may offer an environment that supports not only learning but also psychological adaptation to aging and the maintenance of healthy behaviors.

The quality-of-life findings also support the main results of the study. U3A students had higher CASP-19 total scores and higher scores on both the Perception of autonomy and satisfaction and Perception of barriers subscales. Higher scores on the latter indicate fewer perceived barriers. These findings suggest that participation in lifelong learning programs may be positively associated with how older adults evaluate their lives. Particularly in older age, feeling in control of one’s life, actively participating in decision-making, and maintaining social roles are considered important determinants of quality of life [8, 9]. The social interaction, sense of productivity, and lifelong learning opportunities offered by U3A may be among the factors associated with this finding.

Although previous studies have reported a higher quality of life among older adults participating in lifelong learning programs, the mechanisms underlying this relationship remain insufficiently understood. Ozamiz-Etxebarria et al. (2022) reported a higher quality of life among U3A students [9], whereas Zhang et al. (2024) found that the relationship between educational quality and subjective well-being was largely explained by the fulfillment of autonomy, competence, and relatedness needs [23]. Similarly, Bužgová et al. (2024) reported meaningful improvements in quality of life, life satisfaction, perceived social support, and psychological adaptation among older adults who completed a one-year U3A program [22]. The significant differences observed in both CASP-19 subscales in the present study suggest that lifelong learning may be associated not only with acquiring knowledge but also with psychological needs and life satisfaction.

Importantly, the effects of U3A programs may depend on the content and structure of specific courses and activities. In a recent quasi-experimental study conducted in Türkiye, participation in a 12-week U3A educational program was associated with improvements in quality of life, life satisfaction, and successful aging [24]. These findings suggest that structured educational content may be an important component of U3A programs; however, the relative contribution of specific course types, educational content, and social or recreational activities remains unclear and should be examined in future comparative and intervention studies.

One of the main findings of this study was the moderate positive correlation between healthy aging and quality of life. This finding indicates that the two concepts are related. In particular, the Having Social Relationships and Support, Staying Physically Active, and Staying Cognitively Active and Having Social Participation subscales showed stronger correlations with quality of life. The similar magnitude of these correlations suggests that physical activity showed an association with quality of life comparable to those observed for social relationships and cognitive and social participation in this sample. Physical activity and social engagement may also be interrelated, as group-based physical activities may foster social interaction and reduce social isolation, while greater social support for physical activity has been associated with higher levels of physical activity [29, 31, 32]. Conversely, limitations in physical activity may restrict opportunities for social participation, although this relationship was not directly examined in the present study. Recent studies have reported that social participation is associated with greater life satisfaction, less loneliness, and better psychological well-being [14, 17, 33]. Taken together, these findings suggest that physical activity, social relationships, and cognitive and social participation may represent interrelated dimensions associated with quality of life.

The correlation analysis showed that Having Social Relationships and Support had one of the strongest relationships with quality of life. In older age, both the size and quality of social networks may be related to quality of life. Regular social interaction may reduce loneliness, strengthen coping skills, and support psychological resilience [14, 16, 17]. Social networks may also provide motivation for maintaining healthy behaviors [22]. The opportunities for establishing sustainable social relationships offered by U3A programs may be one possible factor associated with the higher social support and quality-of-life scores observed in our study.

The regression analyses provided another important finding. U3A student status remained significantly associated with both healthy aging and quality of life after adjustment for age, gender, marital status, education level, chronic disease status, self-rated health status, cigarette/tobacco use, and regular health check-ups. This finding suggests that the higher scores observed among U3A students may not be fully explained by the sociodemographic and health-related variables included in the models. The findings indicate that participation in lifelong learning programs may be associated with healthy aging and quality of life after accounting for the variables examined.

The findings may have several implications for public health and services for older adults. In aging societies, healthy aging initiatives often focus on chronic disease management and healthcare delivery. The associations observed in this study suggest that community-based programs supporting social participation and lifelong learning may also be considered within healthy aging initiatives. A recent quasi-experimental study also reported significant improvements in quality of life, life satisfaction, and successful aging following participation in a 12-week U3A program [24]. Different course types or program components may have differential effects on quality of life and healthy aging outcomes; however, this requires further investigation. Healthcare providers, public health professionals, social workers, and other professionals working with older adults may contribute by directing older adults toward opportunities for social participation, lifelong learning, and cognitive engagement alongside disease management. Supporting and improving access to programs such as U3A through collaboration among local authorities, universities, and healthcare institutions could be considered a potential approach to facilitating older adults’ participation and contributing to the development of more comprehensive healthy aging policies and practices.

## Limitations

This study has several limitations. First, the cross-sectional design precludes causal inferences between the variables. Individuals who participated in the program may have had higher motivation, stronger social networks, or better health status at baseline, potentially resulting in selection bias. Second, the use of self-reported data may have introduced recall bias and social desirability bias, which may have affected the accuracy of participants’ responses. Third, the single-center design limits the generalizability of the findings to older populations in other regions. In addition, the use of non-probability sampling and the relatively limited sample size may have affected the representativeness and precision of the findings and reduced the ability to detect modest associations. Furthermore, because the duration of program participation, class attendance, and intensity of engagement in activities were not assessed, the potential dose–response relationship between the level of U3A participation and healthy aging and quality of life could not be examined. Future longitudinal and interventional studies should evaluate these variables and help clarify the temporal and causal relationships between participation in lifelong learning programs and healthy aging and quality of life.

## Conclusions

In conclusion, U3A students had higher levels of healthy aging and quality of life than their peers who were not enrolled in the program, and these differences remained significant after adjustment for the sociodemographic and health-related variables examined. A moderate positive correlation was also found between healthy aging and quality of life, with having social relationships and support, staying physically active, and staying cognitively active and having social participation showing stronger associations with quality of life. Our findings indicate that participation in lifelong learning programs may be positively associated with healthy aging and quality of life. However, causal inferences cannot be made because of the cross-sectional design. Programs such as U3A may be considered community-based healthy aging initiatives; nevertheless, longitudinal and interventional studies are needed to determine their potential contributions.

## Data Availability

The datasets used and/or analysed during the current study are available from the corresponding author on reasonable request.

## Declarations

### Declarations

This manuscript has not been published previously and is not under consideration for publication elsewhere.

## Abbreviations

CASP-19: Quality of Life Scale in Older People
CI: Confidence interval
HAI: Healthy Aging Instrument
SD: Standard deviation
STROBE: Strengthening the Reporting of Observational Studies in Epidemiology
U3A: University of the Third Age
VIF: Variance inflation factor
WHO: World Health Organization
